# Developing the anemone *Aiptasia* as a tractable model for cnidarian-dinoflagellate symbiosis: the transcriptome of aposymbiotic *A. pallida*

**DOI:** 10.1186/1471-2164-13-271

**Published:** 2012-06-22

**Authors:** Erik M Lehnert, Matthew S Burriesci, John R Pringle

**Affiliations:** 1 Department of Genetics, Stanford University School of Medicine, Stanford, CA, 94025, USA

**Keywords:** Anemone, Cnidaria, Coral, Dinoflagellate, Neuropeptide, Single-nucleotide variant, Symbiosis, Transcriptome

## Abstract

**Background:**

Coral reefs are hotspots of oceanic biodiversity, forming the foundation of ecosystems that are important both ecologically and for their direct practical impacts on humans. Corals are declining globally due to a number of stressors, including rising sea-surface temperatures and pollution; such stresses can lead to a breakdown of the essential symbiotic relationship between the coral host and its endosymbiotic dinoflagellates, a process known as coral bleaching. Although the environmental stresses causing this breakdown are largely known, the cellular mechanisms of symbiosis establishment, maintenance, and breakdown are still largely obscure. Investigating the symbiosis using an experimentally tractable model organism, such as the small sea anemone *Aiptasia*, should improve our understanding of exactly how the environmental stressors affect coral survival and growth.

**Results:**

We assembled the transcriptome of a clonal population of adult, aposymbiotic (dinoflagellate-free) *Aiptasia pallida* from ~208 million reads, yielding 58,018 contigs. We demonstrated that many of these contigs represent full-length or near-full-length transcripts that encode proteins similar to those from a diverse array of pathways in other organisms, including various metabolic enzymes, cytoskeletal proteins, and neuropeptide precursors. The contigs were annotated by sequence similarity, assigned GO terms, and scanned for conserved protein domains. We analyzed the frequency and types of single-nucleotide variants and estimated the size of the *Aiptasia* genome to be ~421 Mb. The contigs and annotations are available through NCBI (Transcription Shotgun Assembly database, accession numbers JV077153-JV134524) and at http://pringlelab.stanford.edu/projects.html.

**Conclusions:**

The availability of an extensive transcriptome assembly for *A. pallida* will facilitate analyses of gene-expression changes, identification of proteins of interest, and other studies in this important emerging model system.

## Background

Coral reefs are global resources of great ecological, economic, and aesthetic value. The success of corals in their typically nutrient-poor environments is due largely to their symbiosis with dinoflagellates of the genus *Symbiodinium*. These algae inhabit the symbiosome (a vacuole derived from the early endosome) in gastrodermal cells of the host [1-4] and transfer up to 95% of their photosynthetically fixed carbon to the host [5]. Reef-building corals have recently declined worldwide, with pollution, disease, destructive fishing practices, increased sea-surface temperatures, and ocean acidification all implicated as contributory factors. Some of these environmental changes affect the symbiotic relationship between algae and host and can lead to dramatic and potentially lethal “bleaching” events, during which the algae are lost and the host may die. Bleaching events have become more frequent over the past 20 years.

Much recent research in coral biology has focused on the effects of stresses – particularly high temperature and lowered pH – on the coral holobiont (the community of living organisms making up a healthy coral), as well as on which genetic and molecular factors of the host and alga lead to differential stress responses and resilience [6-13]. However, these efforts have been impeded by the lack of an experimentally tractable system for studies of the establishment, maintenance, and breakdown of the symbiosis. Corals themselves present major logistical difficulties for laboratory investigation. They grow slowly and are difficult and costly to maintain, their calcareous skeletons make many biochemical and cell biological techniques difficult, and it can be difficult to obtain sufficient biomass to do high-throughput experiments. In addition, samples collected from the wild can have heterogeneous genetic backgrounds, causing difficulties in the application and interpretation of gene-expression studies.

To circumvent these difficulties, we and others are developing the small sea anemone *Aiptasia* as a model system for studies of dinoflagellate-cnidarian symbiosis [14,15]. Like corals, *Aiptasia* is an anthozoan (a Class in the Phylum Cnidaria) and maintains intracellular symbiotic dinoflagellates closely related to those in corals. However, unlike corals, *Aiptasia* is extremely hardy, grows and reproduces rapidly via asexual reproduction in the laboratory (allowing the generation of large clonal populations), and lacks a calcareous skeleton. The lack of skeletal deposition makes *Aiptasia* an unsuitable model for this aspect of coral biology but greatly facilitates other studies of cell biology and biochemistry. Additionally, *Aiptasia* can exist in an aposymbiotic (dinoflagellate-free) state or host a variety of *Symbiodinium* types (although not all), allowing facile studies of symbiosis specificity [14,16,17]. We have recently developed a protocol for the year-round induction of spawning and larvae production in laboratory-raised *Aiptasia*[18], which should free a variety of studies from dependence on the seasonal coral reproductive cycle and potentially open the door to genetic analysis.

Studies of the dinoflagellate-cnidarian symbiosis can take advantage of genomics approaches. For example, gene-expression studies should help to elucidate how symbiotic cnidarians respond to various stressors, whereas comparative genomics approaches using sequence data from cnidarians that are not symbiotic with dinoflagellates should help us understand how these symbioses evolved. Genomic and transcriptomic resources for cnidarians are beginning to accumulate rapidly, thanks to the advent of new sequencing technologies. Recently, the genome of *Acropora digitifera*, a common Indo-Pacific coral, was sequenced and assembled [19]. In addition, the genomes of two non-symbiotic cnidarians, the anemone *Nematostella vectensis* (an anthozoan) and the more distantly related *Hydra magnipapillata* (in Class Hydrozoa), have been sequenced [20,21]. Small, Sanger-sequenced EST datasets are available for several species of corals and anemones [15,22,23], as are larger 454-sequenced datasets for several corals [24-26].

As a step in the development of *Aiptasia* as a model system, we have performed a detailed analysis of the transcriptome of the aposymbiotic animals. Unlike previous transcriptomes in the field of symbiotic cnidarian biology, these data are derived from a clonal and easily distributed strain of anemone, greatly facilitating a straightforward comparison of experimental results between different laboratories.

## Methods

### *Aiptasia* strain and culture

All animals used were from clonal population CC7 [15], which in spawning experiments typically behaves as a male (hundreds of spawns have produced sperm compared to three occasions on which individual polyps have produced eggs) [18]. The stock cultures were grown in a circulating artificial seawater (ASW) system at ~25°C with 20–40 μmol photons m^-2^ s^-1^ of photosynthetically active radiation (PAR) on an ~12 h light : 12 h dark cycle and fed freshly hatched brine-shrimp nauplii approximately twice per week. Aposymbiotic animals were generated by several repetitions of the following process: cold-shocking by addition of 4°C ASW and incubation at 4°C for 4 h, followed by 1–2 days of treatment at ~25°C in ASW containing the photosynthesis inhibitor diuron (Sigma-Aldrich) at 50 μM. After recovery for several weeks in ASW in the light (~12:12 light:dark) at ~25°C, putatively aposymbiotic anemones were inspected by fluorescence microscopy to confirm the complete absence of dinoflagellates (whose bright chlorophyll autofluorescence is conspicuous when they are present) and were then cultured in separate tanks as described for the stock culture above.

### RNA extraction and sequencing

Separate populations of animals were exposed to various conditions prior to RNA isolation in an attempt to maximize the diversity of genes expressed. Whole, medium-sized (~1 cm long) anemones were collected in three pools: (i) ~20 animals grown in control conditions; (ii) animals (2–3 per concentration and time point) exposed to bacterial lipopolysaccharide [LPS (Sigma, cat. no. L2880), which is commonly used to induce a strong innate immune response in other organisms] at 1, 10, or 100 μg/μl for 6 or 24 h; (iii) animals (2–3 per treatment) that had been exposed to a single treatment [elevated light (~250 μmol photons m^-2^ s^-1^) for 3 h; dark for 3 h; cold shock at 4°C for 4 h; heat shock at 37°C for 4 h; ultraviolet illumination for several minutes; starvation for one week; hyperosmolarity (1.5x normal salt concentration) or hypoosmolarity (0.3x normal salt concentration) for 30 min; exposure to 10 μM or 100 μM of the 20 standard amino acids or the sugars sucrose and D-glucose for 1 h]. Treated animals were stored in RNALater (Ambion, cat. no. AM7021) at −20°C for later processing.

We extracted total RNA from the anemones in each pool by homogenization in TRIzol reagent (Invitrogen, cat. no. 15596–026) following the manufacturer’s protocol and using the alternative high-salt method of RNA precipitation recommended by Invitrogen to reduce proteoglycan and polysaccharide contamination. We enriched for polyadenylated RNA using the MicroPoly(A) Purist kit (Ambion, cat. no. 1919) and then fragmented the RNA using divalent cations [5 min at 94°C in the reverse-transcriptase first-strand buffer supplied with SuperScript III reverse transcriptase (Invitrogen, cat. no. 18080044)]. cDNA was synthesized using random-hexamer primers (Invitrogen, cat. no. N8080127), ligated to Illumina PE Adaptors, size-selected, amplified, and size-selected a second time. Libraries with different insert sizes (ca. 200, 400, and 600 bp) were synthesized for each pool. Clustering and sequencing were performed by the Stanford Center for Genomics and Personalized Medicine using an Illumina Genome Analyzer IIx (GAIIX) sequencer.

### Read filtering and transcriptome assembly

To minimize redundancy in the dataset, we used the Fulcrum program to collapse duplicate reads and return a single representative read with improved quality scores for each “read family” [27]. Reads were then filtered for quality and length. Briefly, reads were trimmed such that no nucleotide had a quality score less than 10 and no ambiguous nucleotides (N’s) remained. Any read shorter than 45 bp was then discarded. The remaining reads were combined into files based on the insert size of the library (irrespective of the prior biological treatment) and assembled using an additive multiple-*k*-mer (35, 39, 43, 47, 51, 55, 59, 63, and 67) approach [28,29] with the Velvet/Oases assembler (Velvet version 1.1.04 and Oases version 0.1.21) [30].

Oases assembled many contigs that formed “hairpins”, suggesting mis-assembly caused by the presence of palindromic or near-palindromic sequences in the reads. (This problem appears to have been solved in more recent versions of Oases that were released after our study was completed [30].) We identified these hairpin-containing sequences and split each of them into two separate contigs. The contigs resulting from the individual assemblies were then assembled together with the original Illumina reads using a *k*-mer length of 67 with the *conserveLong* option turned on. Both the output from this final assembly and the combined contigs from each individual assembly were merged into a single file, new hairpins were identified and processed as described above, and identical contigs were collapsed into single representatives using cd-hit-est [31]. The resulting contigs were assembled using CAP3 (requiring ≥50-bp overlap with ≥90% identity to join two contigs) to join overlapping contigs and reduce redundancy in the transcriptome dataset [32]. Contigs shorter than 200 bp were discarded as likely to be uninformative.

### Transcriptome annotation

In order to assign putative functional roles to the transcripts, we aligned them to the NCBI non-redundant protein database (nr) using the blastx program from the standalone BLAST 2.2.25+ software suite with an e-value cutoff of 1e-3 [33]. Predicted protein sequences were searched for specific domains using Interproscan [34]. The blastx and Interproscan outputs were imported using the Blast2GO software package [35] and used to assign Gene Ontology (GO) terms to the predicted proteins [36].

### Validation of contigs by alignment with paired-end Sanger reads

As one approach to contig validation, we aligned a set of paired-end Sanger-sequenced ESTs [15] to our transcriptome assembly using BLAT (minimum percent identity 90%) [37]. We counted the number of times the best alignments of a pair of forward and reverse Sanger reads were to the same contig but with the expected opposite orientation.

### Genomic DNA extraction and sequencing

Genomic DNA was isolated from medium-sized aposymbiotic anemones by incubating the whole animals at 55 °C for 4 h in lysis buffer (100 mM NaCl, 50 mM Tris pH 8.0, 50 mM EDTA, 1% SDS) to which Proteinase K had been added to a final concentration of 0.77 μg/μl. The resulting solution was extracted twice with equal volumes of buffer-saturated phenol (Invitrogen, cat. no. 15513–039) and once with an equal volume of phenol/chloroform/isoamyl alcohol (25:24:1). The genomic DNA was then precipitated by ethanol, resuspended in 100 μl of TE buffer, and sheared using a Covaris Adaptive Focused Acoustics machine, following the manufacturer’s instructions, to a target size of 400 bp (10% Duty Cycle, 4 Intensity, 200 cycles per burst, 55 seconds). End-repair and adapter ligation were performed following Illumina’s instructions, and two lanes were sequenced using an Illumina HiSeq system by the Stanford Center for Genomics and Personalized Medicine.

### Heterozygous SNV detection

Putative single-nucleotide variants (SNVs) were detected using CLC Genomics Workbench version 4.6 (CLC bio). Fulcrum-collapsed (see above) and quality-filtered HiSeq genomic reads were mapped against the transcriptome. After an optimal alignment was generated, it was considered valid if 40% of the read aligned with ≥96% agreement (at least 34 of 35 base-pairs for the average post-trimming read length of 88-bp). We used 40% rather than something higher because the 100-bp reads could overlap exon-intron boundaries, and we do not yet have a good estimate of average exon size in *Aiptasia*. The 40% criterion should prevent intron sequence in the read from disallowing a valid match while still providing sufficient specificity. If a given site had a minimum of 10x coverage and ≥35% of the reads at that site contained the alternative base, we classified that base as an SNV.

To estimate the percentage of false positives among our SNV calls, we amplified genomic DNA for some of them using primers to the flanking sequences and sequenced the products using the Sanger method. We identified SNVs as positions in otherwise high-quality chromatograms where there were peaks for two different bases.

### Estimation of genome size

Genome size was estimated by using a slightly modified version of the protocol outlined by Hu *et al.*[38]. We aligned two lanes of HiSeq genomic data (see above) against the assembled transcriptome using BLAT. We determined the number of bases in each read that aligned with the corresponding contig from the top hit that had no alignment gaps; where multiple hits with equal scores existed, the first hit listed was used. The numbers of aligned bases were summed for all genomic reads mapping to a given contig and divided by the contig length, giving each contig in the transcriptome an average coverage. The modal coverage of the entire contig dataset was then used to estimate the depth to which the genome had been sequenced. The total amount of sequence in the genomic reads was then divided by the estimated sequencing depth to obtain the genome size.

## Results and discussion

### Sequencing and assembly of the transcriptome

From a laboratory-raised, clonal population of *Aiptasia pallida*, we generated aposymbiotic anemones and confirmed that they were dinoflagellate-free as described in Methods. We then used these animals to produce three pools of mRNA for cDNA synthesis: mRNA from animals grown in control conditions, mRNA from LPS-treated animals, and mRNA from animals subjected to a variety of other treatments. From each pool of mRNA, we then synthesized three paired-end Illumina libraries with different insert sizes. Those libraries deemed the best quality by Bioanalyzer trace were then sequenced in separate lanes using the Illumina GAIIX system (Table 1). The resulting 10 lanes of sequence yielded a total of ~208 million raw pairs of reads and ~36 Gb of sequence. We used the Fulcrum program [27] on the sequence data from each lane to collapse duplicate sequences due to either PCR amplification or high coverage of abundant transcripts; this operation reduced the number of reads to ~44 million pairs. (The large reduction presumably represented overamplification of the original libraries.) After trimming to remove low-quality and adaptor sequences and removing short reads, the number of reads was further reduced to ~42 million pairs comprising ~7.4 Gb of sequence.

**Table 1:** Properties of the libraries and sequencing runs used for transcriptome analysis

**Population from which mRNA was derived**	**Approximate library insert length (bp)**	**Number of GAIIx cycles (bp)**	**Number of GAIIx lanes sequenced**	**Amount of sequence (Gb)**
Control stock	200	76	3	9.1
Mixed-treatment	600	76	3	12.8
LPS-treated	200	101	1	5.3
LPS-treated	400	101	1	4.9
LPS-treated	600	76	1	2.7
LPS-treated	600	101	1	1.8

**Figure 1:**
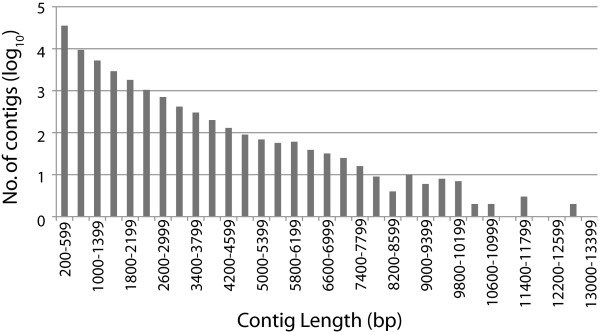
**Distribution of contig lengths in the transcriptome assembly.** The 11000–11399, 11800–12199, 12200–12599, and 13000–12399 ranges each contained one contig (hence log_10_ = 0).

The collapsed, quality-filtered reads were assembled using Velvet/Oases following a multiple-*k*-mer approach (see Methods). The resulting assemblies were merged, resulting in an initial set of 69,402 contigs. Of these, 11,384 appeared to be due to bacterial contamination as judged by their strong similarity to known bacterial sequences. Most of these contigs were derived from the LPS-treatment libraries and probably resulted from the presence of bacterial DNA in the LPS stock. The remaining 58,018 contigs ranged from 200 bp to 13,061 bp, with a mean of 770 bp and an N50 of 1,185 bp (Table 2). Although the size distribution was weighted towards smaller contigs (Figure 1), there were 13,208 contigs with lengths >1,000 bp.

**Table 2:** **Summary of the aposymbiotic*Aiptasia*transcriptome assembly**

Total number of contigs	58,018
Total base-pairs in contigs	44.7 Mb
Contig size range	200–13,061 bp ^a^
Median contig length	453 bp
Mean contig length	770 bp

^**a**^**Contigs of <200 bp were discarded (see text).**

### Validation and functional annotation

We used several approaches to validate the transcriptome assembly. First, we compared it to a set of 4,833 pairs of Sanger-sequence reads from a cDNA library derived from mRNA isolated from symbiotic anemones [15]. In the preparation of this library, an effort was made to obtain full-length cDNAs, which were also size-selected to enrich for longer species (average size ≅ 1.95 kb); thus, it should be enriched for full-length or near-full-length transcripts. When we aligned these ESTs to our transcriptome assembly using BLAT, 73% (7,091) of the Sanger reads mapped to the transcriptome with identity ≥90%. The remaining 27% are likely to be sequences from *Symbiodinium*, from genes that are expressed only at low levels in aposymbiotic *Aiptasia*, or from other organisms that were present in the culture used to prepare the library for Sanger sequencing. Of the 755 Sanger read-pairs in which each read mapped to one and only one contig, 73% (551) mapped to the same contig in opposite directions. Of the additional 1,520 read-pairs with valid alignments in which one or both reads aligned to more than one contig, for 82% (1,239) there was at least one contig to which both reads aligned with opposite orientations. These data suggest that even among long transcripts, which are more likely to be fragmented in our assembly, many are represented by full-length or near-full-length contigs.

To further validate and begin assigning gene functions to the assembled transcripts, we used blastx to align them to the NCBI non-redundant protein database (nr). Using an e-value cut-off of 1e-10, we found that 49.6% (28,794) of the contigs encoded predicted proteins with significant similarity to proteins in nr. In 70.0% (20,154) of these cases, the top hit was to a predicted protein from *Nematostella*. The large number of contigs without nr hits appears to be due mainly to the presence of many short contigs (Table 3), which presumably cover only non-conserved regions in the encoded proteins. The 28,794 contigs with nr hits identified only 14,479 unique protein accessions. This may be due both to the presence of multiple alternative contigs produced by Velvet/Oases for many transcripts and to the alignment of shorter contigs to different parts of the same protein. The presence of contigs that represent only a portion of a transcript, particularly from those transcripts that are expressed at low levels, makes it difficult at this time to achieve full clustering of contigs derived from alternative transcripts of the same gene (or from different regions of the same transcript), as well as to determine the number of alternative transcripts produced by each genetic locus. Using Blast2GO, we assigned GO terms based on the transcripts’ associated nr annotations – using the default e-value cutoff of the Blast2GO software, 1e-3 - and the results of InterProScan [39]. We were able to assign GO terms to 14,904 contigs in our transcriptome.

**Table 3:** Length dependence of BLAST alignment success

Contig size range	**Total number of contigs in size range**	**Number of contigs in size range with BLAST alignments (e-value ≤ 1e-10)**	**% of contigs in size range with BLAST alignments (e-value ≤ 1e-10)**
200 bp – 599 bp	35,424	11,356	32%
600 bp – 999 bp	9,372	6,080	65%
1000 bp – 1399 bp	5,239	4,113	79%
≥ 1400 bp	7,983	7,244	91%

We also looked for complete or partial coding sequences of conserved genes in several cellular pathways. In one analysis, we examined the proteins predicted from the *Nematostella* genome sequence to be involved in eight metabolic pathways. Sequences assigned to these pathways were downloaded from the Kyoto Encyclopedia of Genes and Genomes (KEGG) [40], and their putative orthologs in the *Aiptasia* transcriptome were identified by a best-reciprocal-blast approach (Table 4). These results suggest that our transcriptome provides complete or nearly complete coverage of many pathways and that most *Nematostella* proteins have orthologs that are represented by full-length or nearly full-length transcripts in our *Aiptasia* transcriptome.

**Table 4:** **Estimating transcriptome completeness by comparison to*Nematostella***^**a**^

**Pathway**	**Number of predicted proteins assigned to this pathway in*Nematostella***	**Number of orthologs in*Aiptasia*transcriptome**	**Average% of*Nematostella*coding sequence covered by best alignment**	**Average predicted amino acid similarity (%)**^**b**^
Glycolysis and gluconeogenesis	30	25	89	87
Amino-sugar and nucleotide-sugar metabolism	28	25	68	85
Regulation of autophagy	13	10	63	85
Pentose-phosphate pathway	18	14	81	89
Citrate cycle	28	24	90	89
Valine, leucine, and isoleucine degradation	37	34	80	88
Purine metabolism	92	78	62	86
Fatty acid biosynthesis	5	3	62	79

^**a**^*Aiptasia* orthologs to *Nematostella* proteins in each pathway (as identified in KEGG) were predicted by best-reciprocal-BLAST analysis (see text).

^b^ Using BLOSUM62 similarity matrix [41].

In a second analysis, we looked individually at the *Aiptasia* homologues of an arbitrarily chosen functional group of proteins, namely a subset of those involved in cellular spatial organization and cytoskeletal function. The results (Table 5) show clearly (i) that many *Aiptasia* genes are represented in our transcriptome assembly by contigs that cover the entire coding region plus sequences of the 5′- and 3′-UTRs; (ii) that even some very long transcripts are represented by contigs that cover most of their lengths; (iii) that, for whatever reason(s), even some genes of moderate length are not represented by complete transcripts in our current assembly (see the septin entry in Table 5); and (iv) that, as expected, proteins in this functional group are closely conserved in *Aiptasia* as they are in other animals.

**Table 5:** Completeness of transcripts and sequence conservation for some proteins involved in cellular spatial organization

**Query protein (GenBank Accession Number)**	**Amino acids in query sequence**	**Amino acids of query covered by best BLAST hit(s)**	**% amino-acid sequence identity (number of gaps)**	**Length of*Aiptasia*contig (bp)**	**Positions in contig covered by best BLAST hit**
Mouse Cdc42 (P60766)	191	1-191	92 (0)	1,399	275-847
Mouse cyto-plasmic actin 1 (P60710)	375	2-375	97 (0)	1,455	117-1,247
Mouse tubulin α1B (P05213)	451	1-432	97 (0)	1,971	1,879-584
Mouse tubulin β5 (P99024)	444	1-427	98 (0)	2,592	1,529-249
Mouse septin-2 (P42208) ^a^	361	139-329	65 (14^b^)	1,979	3-617
Mouse kinesin 1 heavy chain (Q61768)	963	3-199	72 (0)	906	592-2
		453-947	53 (14)	2,138	6–1,514
Mouse myosin 8 (P13542)	1,937	23-1,903	50 (14)	7,798	7,650-2,032
Mouse dynein heavy chain 1 (Q9JHU4)	4,644	21-216	53 (4)	790	191-790
		836-4,643	73 (30)	11,600	3–11,414

^a^ Our unpublished studies have shown that as expected, the *A. pallida* genome encodes multiple septins and that, like other septins [42], these contain the three conserved motifs of a GTP-binding site in their N-terminal regions. We do not yet know why none of these transcripts appears in full-length form in the current transcriptome.

^b^ The predicted *A. pallida* protein has a single insertion of 14 amino acids near the C-terminus of the “septin-unique element” [42].

In summary, although we are undoubtedly lacking the sequences (or at least lacking complete sequences) for some transcripts that are expressed only at low levels, in particular cell types, during particular stages of development, or under conditions to which we did not expose the anemones, it appears that the transcriptome described here contains at least partial sequences (and many full-length sequences) for the large majority of transcripts expressed in adult, aposymbiotic anemones. It will be particularly interesting to see how many additional transcripts are identified when the transcriptome of symbiotic anemones is examined.

### Estimation of SNV frequency

We identified SNVs (*i.e.,* sites of heterozygosity in our clonal *Aiptasia* stock) by mapping genomic data to the transcriptome (see Methods). To minimize the misclassification of sequencing errors as SNVs, we demanded that any SNV called be represented in ≥35% of the reads mapping to a region. We identified 48,404 putative SNVs (not including deletions and insertions) by mapping one lane of HiSeq genomic data (~5.5 Gb of sequence, of which ~1.3 Gb mapped to the transcriptome), and an additional 6,896 by adding a second lane of genomic data (for a total of ~10.1 Gb, of which ~2.2 Gb mapped to the transcriptome), for a total of 55,300, or 1 SNV per 808 bp. Because the additional lane roughly doubled the amount of sequence mapped but led to only an ~14% increase in the SNVs discovered, further mapping would presumably find few additional SNVs within our clonal strain of *Aiptasia*. The majority of SNVs we identified were transition rather than transversion mutations (Table 6), consistent with findings in other organisms [43] and with the previous observations for *Aiptasia*[15]. Additional investigation using similar methods led to the identification of 8,691 putative deletion or insertion variants (Table 6).

**Table 6:** SNV and indel distributions in Aiptasia

**Variant type**	**Frequency in Transcriptome**
A/G Transition SNV	19,806
C/T Transition SNV	20,087
A/C Transversion SNV	6,846
G/C Transversion SNV	4,440
A/T Transversion SNV	7,408
G/T Transversion SNV	6,232
Insertion or Deletion	8,691 ^a^ [1, 5,773; 2, 1,289; 3, 862; 4, 372; 5, 165; 6,143; 7, 57; 8, 30]

^a^ Broken down within the brackets by the numbers of nucleotides involved.

To evaluate the reliability of our SNV calls, we designed primers to nine contigs in our assembly based on the following criteria. (1) The top BLAST hit was to a cnidarian, so we could be confident that we were looking at an *Aiptasia*-derived contig. (2) The predicted SNV was not located so close to an end of the contig that it would be within 40 bp of the primer that we were using to amplify (as this could have led to confusion from low-quality sequence near the primer sites). (3) The variant was a simple base-pair change rather than an indel (as these would have been undetectable by our method of inquiry). (4) Contigs with multiple SNVs were preferred as this enabled to us perform more tests with fewer primers.

For the nine contigs, we created 12 primer pairs that would amplify regions containing a total of 17 putative SNVs. Of these 12 primer pairs, eight produced clear PCR products with single bands, encompassing a total of 11 putative SNVs. Six of these bands had the predicted sizes, and two were larger (~400 instead of 250 bp and ~1600 instead of 576 bp), presumably indicating the presence of introns. The remaining four primer pairs presumably either needed additional optimization of the PCR reactions to ensure specificity or represented regions in which the exons were separated by introns that were too long for amplification under standard PCR conditions. All eight of the PCR products were sequenced using the same primers as used for the PCR, and the SNV was considered to be validated when there were dual peaks matching the reference and variant calls at the specified location surrounded by otherwise high-quality peaks. All 11 of the SNVs tested were validated in this test, suggesting that there is only a low false-positive rate for our larger set of SNV calls.

### Estimation of genome size

We aligned ~10.1 Gb of genomic reads to the transcriptome assembly (see Methods), and estimated a modal coverage of ~24x per contig. Thus, we estimate a genome size of 10,100 Mb/24 = 421 Mb. *Nematostella* and *Acropora digitifera,* the closest relatives of *Aiptasia* whose genomes have been sequenced, have genome sizes of ~450 Mb and ~420 Mb, respectively [19,20]. Given its apparently modest size, the *Aiptasia* genome could be readily sequenced using the currently available technologies.

### Identification of possible neuropeptide precursors

**Figure 2:**
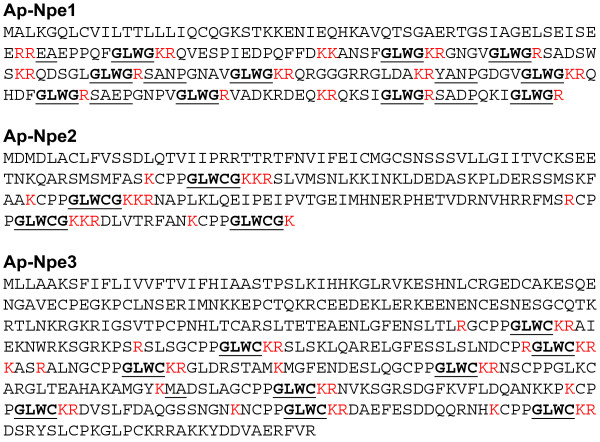
**Predicted amino acid sequences of putative neuropeptide precursors.** The transcriptome was scanned for contigs whose predicted translation products contained multiple copies of the tripeptide GLW, as found in many neuropeptides (see text). Bold face and underlined, GLW motifs plus the immediately following G, CG, or C residues; bold face without underlining, the immediately preceding CPP sequences present in many of these putative peptide precursors; underlining, XA and/or XP dipeptides immediately C-terminal to the potential cut sites (where present); red, basic residues that might direct endoprotease action.

Investigation of neuropeptide precursors and their cleavage products in other cnidarians has improved understanding of their neurological organization and development [44]. It has also provided tools for manipulation of the animals, such as increasing the rate of polyp budding and inducing larval metamorphosis and settlement [44-47]. Attempts to induce settlement of larvae using the *Hydra* neuropeptide Hym-248 (pEPLPIGLW-NH_2_) were successful in several species of the coral genus *Acropora*[46,47] but not in other coral genera [47] or in *Aiptasia* (S. Perez, personal communication). To ask if this failure were due to a lack of sequence similarity between Hym-248 and the neuropeptides in *Aiptasia*, we scanned the transcriptome for potential neuropeptide precursors of the GLW-NH_2_ type. We found three distinct transcripts containing repeated GLW motifs (Figure 2). Interestingly, the three putative precursors differ in the amino acids found immediately downstream of the GLW motif. In Ap-Npe1, this is in all 10 cases a G, suggesting that the mature peptides would indeed terminate in GLW-NH_2_[44], but in Ap-Npe2, each of the four GLW motifs is followed by a CG, suggesting that the mature peptides might terminate in GLWC-NH_2_ (Figure 2). In Ap-Npe3, each of the nine GLW motifs is again followed by a C, but without a G to suggest C-terminal amidation of the mature peptides (or perhaps that this polypeptide is not actually processed to neuropeptides). Each of the putative peptides is also preceded and followed by basic residues that could serve as cutting sites for endoproteases [44], and in some cases the possible endoprotease cut sites are followed by XA and/or XP sequences that could be subject to removal by typical dipeptidylaminopeptidases [44]. Importantly, none of the peptides that might be derived from these putative precursors would be a match either for Hym-248 or for Metamorphosin-A (pEQPGLW-NH_2_, where the N-terminal pyroE is derived from a Q in the primary sequence), a morphogenesis-inducing peptide from the anemone *Anthopleura elegantissima*[44,45], suggesting that the neuropeptide(s) responsible for morphogenesis and induction of settlement differ among cnidarians.

## Conclusions

We have assembled and characterized a reference transcriptome for adult, aposymbiotic *Aiptasia pallida* using the Illumina sequencing platform*.* We have used this resource to detect SNVs in our clonal population of anemones, estimate the genome size, and identify possible neuropeptide-encoding genes. This transcriptome will enable future studies to explore the changes in gene expression that accompany the association with dinoflagellate endosymbionts, determine how the symbiotic partners respond to a variety of stressors, further test the applicability of this model system to corals, and complete the assembly and annotation of the *Aiptasia* genome (for which the transcriptomic data will be essential). The contigs and their associated annotations are available through NCBI (Transcription Shotgun Assembly database, accession numbers JV077153-JV134524) and at http://pringlelab.stanford.edu/projects.html. The limitations of the current assembly should diminish in updated versions that incorporate additional sequence data, particularly those from symbiotic animals and from different developmental stages. Updated assemblies will be made available through both the NCBI site and our lab website.

## Abbreviations

SNV, Single-nucleotide variant; TE, Tris-EDTA; GAIIx, Genome Analyzer IIx; GO, Gene Ontology; PAR, Photosynthetically active radiation; ASW, Artificial seawater; LPS, Lipopolysaccharide; Nr, Non-redundant database; EST, Expressed sequence tag.

## Authors’ contributions

EML and JRP conceived of and designed the project. EML prepared libraries and performed most of the analyses. MSB developed the Fulcrum read collapser and participated in the evaluation and optimization of assembly methods. EML, JRP, and MSB wrote the manuscript. All authors read and approved the final the manuscript.
